# The relationship between social isolation, depression, and cognition: The mediation analysis of depression

**DOI:** 10.1093/geroni/igaf122.2791

**Published:** 2025-12-31

**Authors:** Fanghong Dong, Yeji Hwang

**Affiliations:** Washington University in St. Louis, St. Louis, Missouri, United States; Seoul National University, Seoul, Republic of Korea

## Abstract

Social isolation is a key factor leading to cognitive decline among older adults. However, the underlying mechanisms driving this relationship remain unclear. Given the strong link between social isolation and mental health, this study aimed to examine whether depression mediates the longitudinal relationship between social isolation and cognition. This study was a 12-year longitudinal study using data from the Health and Retirement Study (2006/2008 (T1), 2010/2012 (T2), 2014/2016 (T3), 2018/2020 (T4)). Participants who were 65 years and older were selected (N = 946). Depression was assessed using the Center for Epidemiologic Studies Depression scale. Cognition was assessed via the Telephone Interview for Cognitive Status. Social isolation was measured using 9 items on contact frequency with children, family members, and friends. The longitudinal mediation model was used adjusting for age, gender, race, and education, which demonstrated excellent fit (CFI = 0.990, TLI = 0.986; RMSEA = 0.025, SRMR = 0.022). Higher baseline social isolation significantly predicted elevated baseline depression (β = 0.20, p < .001), which in turn negatively impacted baseline cognition (β = -0.16, p < .001). A significant indirect effect confirmed mediation at the intercept level (β = -0.032, p = .002), while social isolation retained a direct negative effect on cognition (β = -0.10, p = .018). This supported depression as a partial mediator at baseline levels. However, longitudinal slope paths were non-significant, suggesting no dynamic mediation over time. Future studies need to be conducted on identifying the longitudinal mechanism underlying the relationship between social isolation and cognitive decline.

